# The Third-Time Recurrence of a Thoracic Solitary Fibrous Tumor in a Patient Presenting With Paraneoplastic Hypoinsulinemic Hypoglycemia (Doege-Potter Syndrome)

**DOI:** 10.7759/cureus.60256

**Published:** 2024-05-14

**Authors:** Rida Zakar, Nader Saad, Karim Nehme, Antoine El Sett, Pierre El Sett, Georges Tabet

**Affiliations:** 1 Medicine, Université Saint Joseph, Beirut, LBN; 2 Thoracic and Vascular Surgery, Hôtel-Dieu de France, Beirut, LBN; 3 Vascular Surgery, Hôpital Paris Saint Joseph, Paris, FRA; 4 Surgery, Hôtel-Dieu de France, Beirut, LBN

**Keywords:** tumor recurrence, thoracic malignancy, solitary fibrous tumor (sft), doege-potter syndrome, pulmonary lobectomy

## Abstract

We present a case of a 76-year-old Caucasian female with a recurrent solitary fibrous tumor (SFT) of the pleura, showcasing a rare manifestation of hypoglycemia associated with Doege-Potter syndrome (DPS). Having undergone two previous surgeries for SFT, the patient presented to the emergency department with severe fatigue, recurrent episodes of loss of consciousness, and hypoglycemia, despite lacking a history of diabetes mellitus. Radiological evaluation revealed a substantial recurrent SFT in the left lung, prompting excision through a left posterolateral thoracotomy. Remarkably, the patient’s altered mental status and hypoglycemia resolved postoperatively. The case meets the criteria for aggressive SFT behavior, warranting consideration for adjuvant radiation therapy to control the risk of recurrence. This report highlights the nuanced interplay between SFT recurrence, paraneoplastic syndromes like DPS, and the potential benefits of adjuvant therapeutic strategies in managing these complex clinical scenarios.

## Introduction

Solitary fibrous tumors (SFTs) are rare benign or malignant mesenchymal cell tumors; while they can occur anywhere in the body [1,2], they are mainly detected in the visceral pleura [3]. Recurrence is diagnosed in 4% of benign SFTs and 31.2% of the malignant variant of these tumors [4]. SFTs are rarely associated with paraneoplastic syndromes such as those due to insulin-like growth factors responsible for severe hypoglycemia [5]. The occurrence of hypoglycemia with an intrathoracic tumor was first described by Doege and Potter in 1930, and this condition has hence been termed Doege-Potter syndrome (DPS) [6]. DPS is a rare paraneoplastic condition associated with SFTs, presenting as a secretion of a prohormone of insulin-like growth factor II, causing hypoinsulinemic hypoglycemia [3]. Surgery is the most effective treatment for primary and recurrent benign and malignant SFT [7]. Radiotherapy can also be employed in cases with a large tumor, exhibiting positive margins, or those considered malignant SFTs. Furthermore, multiple factors can be considered to orient the surgeon's decision-making. We discuss a recurrent case of a suspected malignant SFT of the pleura presenting with hypoinsulinemic hypoglycemia and explore factors that would lead to a better understanding of disease management and outcomes in this patient population.

## Case presentation

**Figure 1:**
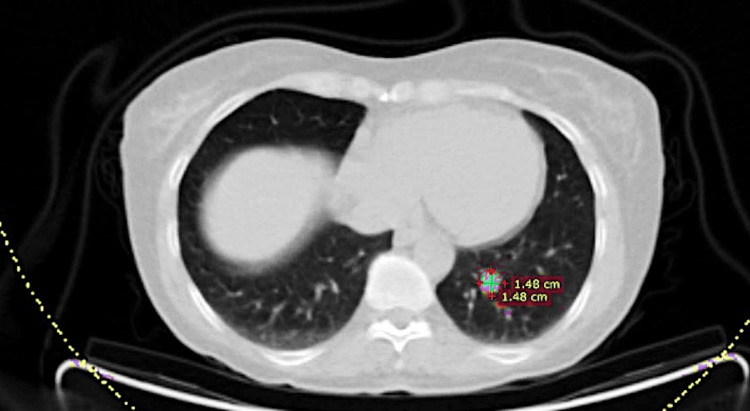
Axial view of the CT scan showing the SFT in the left lower lobe in 2010 CT: computed tomography; SFT: solitary fibrous tumor

**Figure 2:**
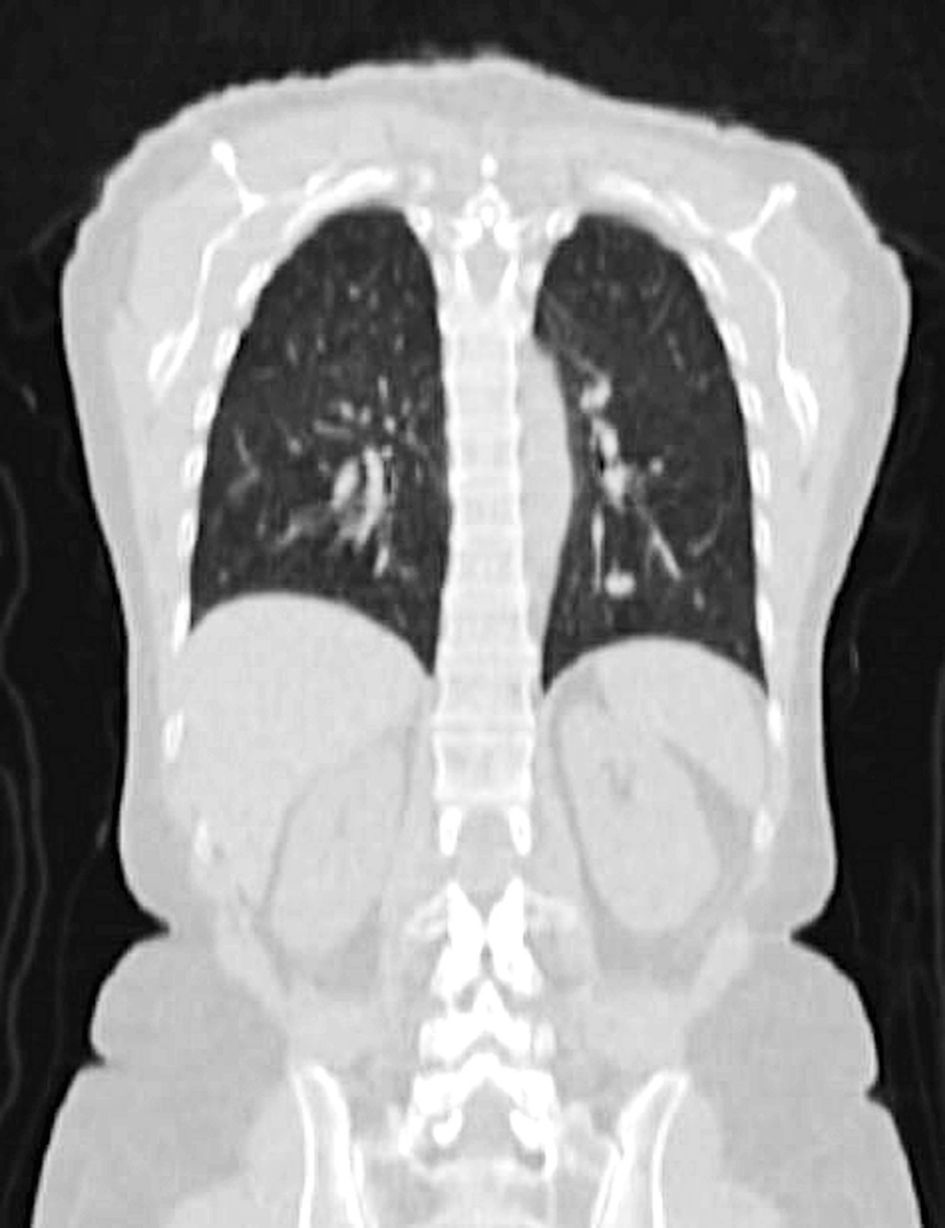
Coronal view of the CT scan showing the SFT in the left lower lobe in 2010 CT: computed tomography; SFT: solitary fibrous tumor

**Figure 3:**
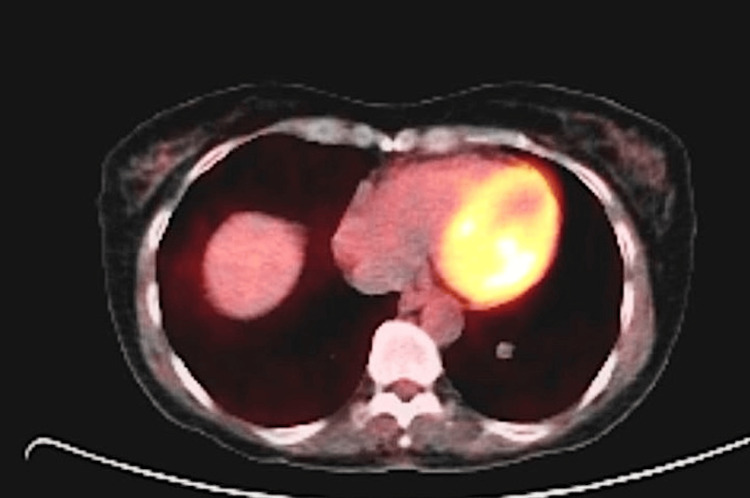
PET scan showing the SFT in the left lower lobe in 2010 PET: positron emission tomography; SFT: solitary fibrous tumor

A 76-year-old Caucasian female presented to the emergency unit due to severe fatigue, lethargy, and loss of appetite. In the past two months, the patient had experienced several episodes of loss of consciousness and hypoglycemia despite not having any known history of diabetes mellitus. She also reported unintentional weight loss of 5 kg during the previous month. Her medical history included a solitary fibrous tumor diagnosed 10 years ago for which she had undergone a segmental resection of the left lower lobe with clear resection margins on pathology (Figures 1-3).

**Figure 4:**
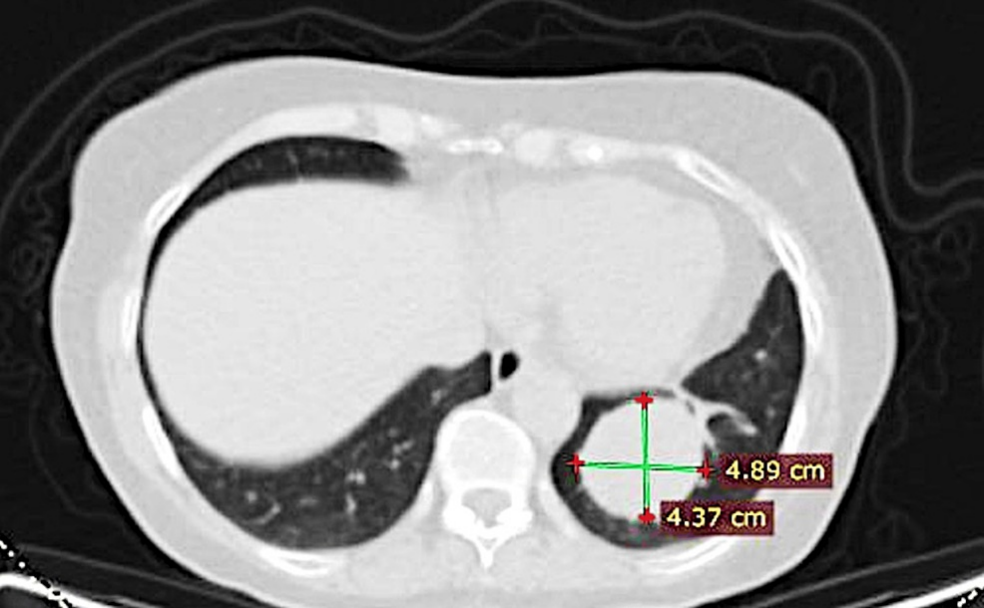
CT scan showing the SFT in the left lower lobe in 2013 CT: computed tomography; SFT: solitary fibrous tumor

However, a follow-up CT scan performed three years later had shown a recurring SFT in the left lower lobe (Figure 4) and a second surgical resection with lobectomy had been performed, with clear resection margins on pathology.

Upon her arrival at the emergency department, the patient had an altered mental status and was severely confused. Initial blood glucose level showed severe hypoglycemia of 28 mg/dl. Cerebral MRI ruled out any neurological cause of her symptoms. The patient’s neurological status returned to baseline following the administration of IV dextrose.

**Figure 5:**
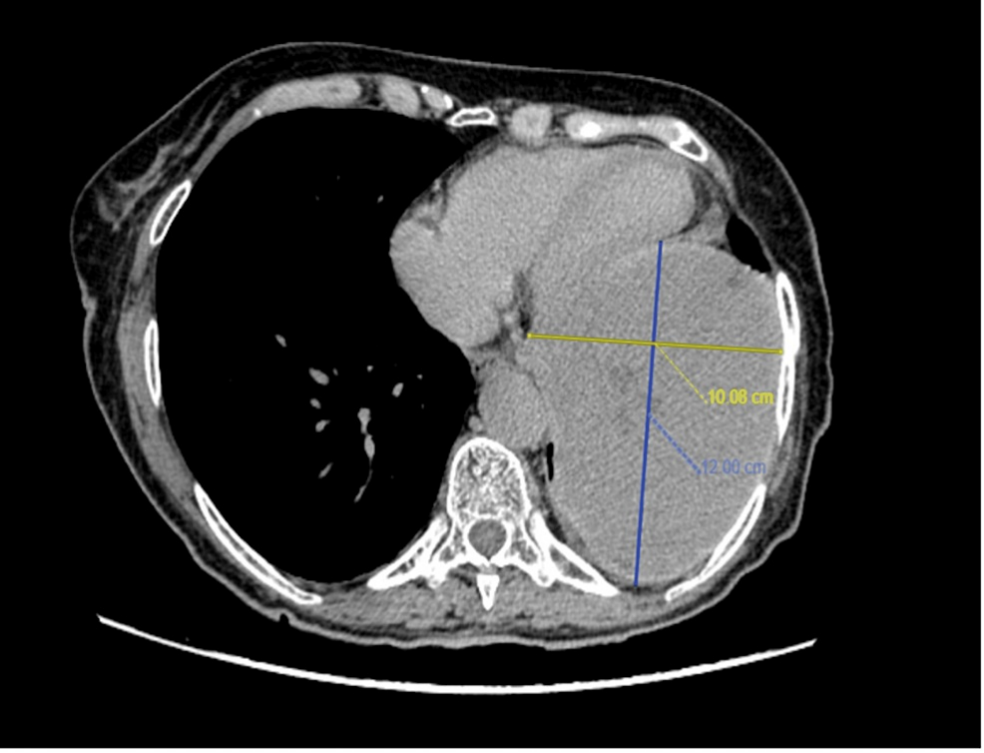
Axial view of a thoraco-abdomino-pelvic scan showing a 12 x 10 cm mass of the left lower lobe in 2021

A subsequent thoracic CT scan showed a 12 x 10 cm heterogeneous mass on the left base of the lung with severe compression of the left ventricle associated with small nodules reaching 9 mm in the aortic and paratracheal regions (Figure 5).

**Figure 6:**
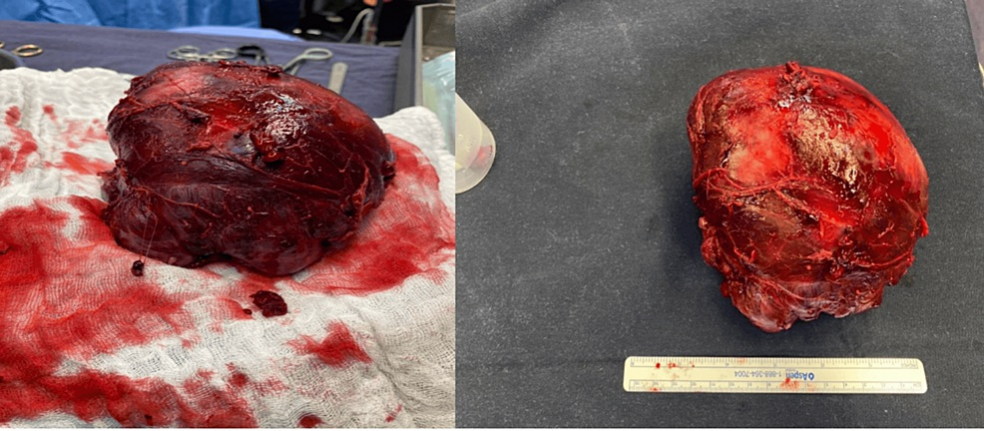
Postoperative images of the resected pleural mass

Based on her latest clinical presentation, radiological evidence of pulmonary mass, and history of recurrent SFT, a biopsy was deemed unnecessary to confirm the diagnosis of DPS. Complete excision of the pleural mass was performed through a left posterolateral thoracotomy with neoplastic cells reaching the limit of excision margins on pathology. The resection specimen can be seen in Figure 6.

The latest pathology specimen (2021) showed a homogeneous hypercellular proliferation, mostly well-limited and encapsulated. Mitoses were estimated at 7 mitoses per high-power field. Necrotic areas were surrounded by fibrous tissue. Tumor cells had infiltrated the vascular walls and, in some places, tumor vascular emboli were noted. The tumor was mostly well-limited but became irregular and infiltrated the capsule in some places. The peritoneum and the diaphragm muscle were adherent to the tumor. 

An immunohistochemical study revealed that the tumor exhibited heterogeneous positivity for CD34, CD99, and desmin. Rare interstitial cells were positive for CD10. Exceptional cells were positive for actin. Cytokeratin, h-Caldesmon, and myogenin were negative. The proliferation index (Ki-67) was high, estimated at 30%. Additionally, a resected pulmonary nodule and para-aortic mass showed similar aspects, suggestive of two metastatic ganglia with capsular effraction. A biopsy before the tumor resection had also revealed intense Bcl-2 positivity.

No postoperative complications were noted in the patient, and her blood glucose level returned to normal several hours post-resection. The patient was discharged in good condition without any notable complaints and referred to radiation therapy.

## Discussion

The England criteria [8] are used to help differentiate benign from malignant SFT, and they include multiple variables such as tumor size, high cellularity, pleomorphism with cytonuclear atypia, more than 4 mitoses per 10 high-power fields and associated necrotic or hemorrhagic areas. Our patient fulfilled four out of five of the above-listed criteria, putting her at high risk of recurrence.

Other criteria have been proposed by many authors such as Gold et al., which include recurrent tumor, macro- or microscopically positive resection margin, size greater than 10 cm, more than 4 mitoses per 10 high-power fields, increased nuclear pleomorphism, increased cellularity, and presence of malignant components [7]. Demicco et al. have suggested similar criteria, which include age greater than or equal to 55 years, size greater than or equal to 15 cm, 4 or more mitoses per 10 high-power fields, and tumor necrosis [9]. Witkin et al.'s criteria include included a size greater than or equal to 10 cm, hypercellularity, more than 1 mitosis per 10 high-power fields, tumor necrosis, or hemorrhage [10].

Complete anatomical resection was technically risky in our patient because of the large size of the tumor and its location. Moreover, Herrmann et al. have noted that 77% of patients presenting with paraneoplastic hypoglycemia will have a malignant outcome with a poor prognosis [5]. Adjuvant therapy is therefore often the preferred option in these patients. The indications and effectiveness of adjuvant radiation in patients with positive margins or recurrent tumors have been debated, and the benefit of radiation therapy remains undetermined [11]. A study conducted by P. Schöffski et al. showed that the local recurrence rate in patients receiving adjuvant radiotherapy (7.1%) was lower than in those not receiving it (26.7%) [12]. This supports the use of adjuvant radiotherapy to prevent local recurrence. Since it was the third recurrence of SFTs in our case, with positive margins at biopsy, radiotherapy was initiated to minimize the risk of future recurrence.

Furthermore, as suggested by Machado et al. [13], additional testing can be performed to further predict patient outcomes and guide management. In this particular study, genetic analysis with the search of NAB2/STAT6 gene fusion and TERT and TP53 mutations was done. Additionally, other biomarkers such as APAF-1 were sought. By comparison, CD34, CD99, and Bcl-2 positivity in this case adds to the present evidence and could perhaps help in risk stratification in these patients.

SFTs are rare slow-growing pleural tumors and are benign in most cases. Malignant behavior is more frequent in patients presenting with paraneoplastic hypoglycemia and those with recurrent tumors, large sizes, or those meeting other histological criteria. Adjuvant radiotherapy can be considered to reduce recurrence in these cases, especially for patients with positive surgical margins.

## Conclusions

This case report contributes to our understanding of SFTs and their potential to cause rare paraneoplastic syndromes such as DPS. The successful surgical resection of the recurrent SFT and the resolution of the patient's hypoglycemia highlight the importance of considering SFTs in the differential diagnosis of hypoglycemia, especially in patients with a history of SFT. The report also emphasizes the potential benefits of adjuvant radiation therapy in controlling aggressive SFT behavior.
